# Analysis of Neurodevelopmental Disorders in Offspring of Mothers With Eating Disorders in Sweden

**DOI:** 10.1001/jamanetworkopen.2021.43947

**Published:** 2022-01-18

**Authors:** Ängla Mantel, Anne K. Örtqvist, Angelica Lindén Hirschberg, Olof Stephansson

**Affiliations:** 1 Division of Clinical Epidemiology, Department of Medicine Solna, Karolinska Institutet, Karolinska University Hospital, Stockholm, Sweden; 2 Theme Women’s Health, Department of Obstetrics, Karolinska University Hospital, Stockholm, Sweden; 3 Department of Obstetrics and Gynecology, Visby County Hospital, Visby, Sweden; 4 Division of Neonatology, Obstetrics and Gynecology, Department of Women’s and Children’s Health, Karolinska Institutet, Stockholm, Sweden; 5 Department of Gynecology and Reproductive Medicine, Karolinska University Hospital, Stockholm, Sweden

## Abstract

**Importance:**

Despite indices of impaired neurodevelopment in children of mothers with eating disorders, it remains unclear whether these children are at increased risk of developing neuropsychiatric diseases.

**Objective:**

To evaluate the association between maternal eating disorders, whether preexisting or ongoing during pregnancy, and offspring neuropsychiatric disease risk.

**Design, Setting and Participants:**

This population-based prospective cohort study used the Swedish Medical Birth Registry and identified singleton births registered between from January 1, 1990, and December 31, 2012. Children of exposed mothers with eating disorders were matched with comparator children of mothers without diagnoses of eating disorders. To adjust for unmeasured shared familial factors, a cluster of exposed children with full maternal cousin comparators was identified. Follow-up was completed on December 31, 2017. Data were analyzed from August 31, 2020, to April 30, 2021.

**Exposures:**

Maternal eating disorder diagnosis.

**Main Outcomes and Measures:**

All children were followed up from 1 year of age for autism spectrum disorder (ASD) and from 3 years of age for attention-deficit/hyperactivity disorder (ADHD). The relative risk of ASD and ADHD was assessed among exposed children, stratified by eating disorder subtype and ongoing vs previous disease, adjusted for potential confounders, including parental socioeconomic status and comorbidities.

**Results:**

Among the 52 878 children included in the analysis, maternal eating disorder exposure (n = 8813) was associated with an increased risk of ADHD (hazard ratio [HR] for anorexia nervosa, 1.42 [95% CI, 1.23-1.63]; HR for bulimia nervosa, 1.91 [95% CI, 1.43-2.54]; and HR for unspecified eating disorder, 2.00 [95% CI, 1.72-2.32]) and ASD (HR for anorexia nervosa, 2.04 [95% CI, 1.58-2.63]; HR for bulimia nervosa, 2.70 [95% CI, 1.68-4.32]; and HR for unspecified eating disorder, 1.95 [95% CI, 1.49-2.54]). After adjustment for parental confounders, the risk of ADHD remained significantly increased, whereas the risk of ASD in children to mothers with bulimia nervosa was no longer significant. Ongoing anorexia nervosa was associated with a significantly higher risk of ADHD (HR, 2.52 [95% CI, 1.86-3.42]) and ASD (HR, 3.98 [95% CI, 2.49-6.27]) compared with previous disease (HRs, 1.26 [95% CI, 1.06-1.48] and 1.81 [95% CI, 1.38-2.38], respectively). Results based on the family cluster were similar to those of the main analysis for maternal exposure to anorexia nervosa and bulimia nervosa.

**Conclusions and Relevance:**

These findings suggest that children born to mothers with eating disorders, in particular disorders that were active during pregnancy, were at increased risk of developing ADHD and ASD. The association could not be fully explained by parental psychiatric comorbidities, and among children of mothers with anorexia nervosa and bulimia nervosa, it could not be explained by unmeasured familial confounding.

## Key Points

**Question:**

Are children of mothers with eating disorders at increased risk of developing neuropsychiatric diseases?

**Findings:**

In this nationwide cohort study of 52 878 children, including 8813 children born to women with an eating disorder and 44 065 matched children born to women without an eating disorder, maternal eating disorder was significantly associated with attention-deficit/hyperactivity disorder and autism-spectrum disorder in offspring.

**Meaning:**

These findings highlight the importance of clinical awareness and intensified support to women with eating disorders and their children and for future research efforts to identify underlying mechanisms of the association.

## Teaser

This cohort study investigates the association between maternal eating disorders and risk of neurodevelopmental disorders in children of mothers with eating disorders using large-scale population-based Swedish health care registers.

## Introduction

Eating disorders are complex and severe psychiatric disorders characterized by an irrational fear of obesity in combination with weight-controlling behaviors resulting in physical complications and impaired psychosocial function.^1^ Nine of 10 individuals affected by eating disorder are women, and the incidence is highest in adolescence and early adulthood,^2^ signifying the importance to understand how the course of eating disorders is affected by pregnancy and vice versa, and not least how it affects the future health of children of mothers with eating disorders.^3^ The disease course is chronic for approximately one-third of women with anorexia nervosa and bulimia nervosa,^4^ and pregnancy constitutes a vulnerable time period that might be associated with an increased risk of relapse among women with a history of an eating disorder.^5,6^ In fact, both ongoing and previous maternal eating disorders have been associated with an increased risk of several pregnancy and neonatal complications.^5,7,8,9,10,11^

In addition to the direct effect on the fetal growth and development, the intrauterine environment presumably influences health during childhood and throughout life.^12,13^ Precise mechanisms and interactions are poorly defined, but it is assumed that intrauterine environmental exposures, including nutritional factors, affect neurodevelopment and immune maturation. Thus, hypothetically, children of mothers with eating disorders might be prone to develop specific conditions, including neurodevelopmental disorders. According to a few studies,^14,15^ children of mothers with an eating disorder are at increased risk of impaired neuropsychiatric and cognitive development and behaviors indicative of psychiatric diseases. Importantly, these previous studies are heterogenous in nature and therefore difficult to compare. Moreover, exposures and outcomes have typically been identified using questionnaires, inventories, or clinical interviews, making them prone to recall bias, and information on potential confounders is generally not available. The aim of this study was to investigate the association between maternal eating disorders and risk of neurodevelopmental disorders in the children using large-scale population-based Swedish health care registers.

## Methods

This cohort study was approved by the Swedish Ethical Review Authority, which waived the need for informed consent owing to use of pseudonymized data. The study followed the Strengthening the Reporting of Observational Studies in Epidemiology (STROBE) reporting guideline.

### Study Setting, Data Sources, and Study Base

**Figure 1.**
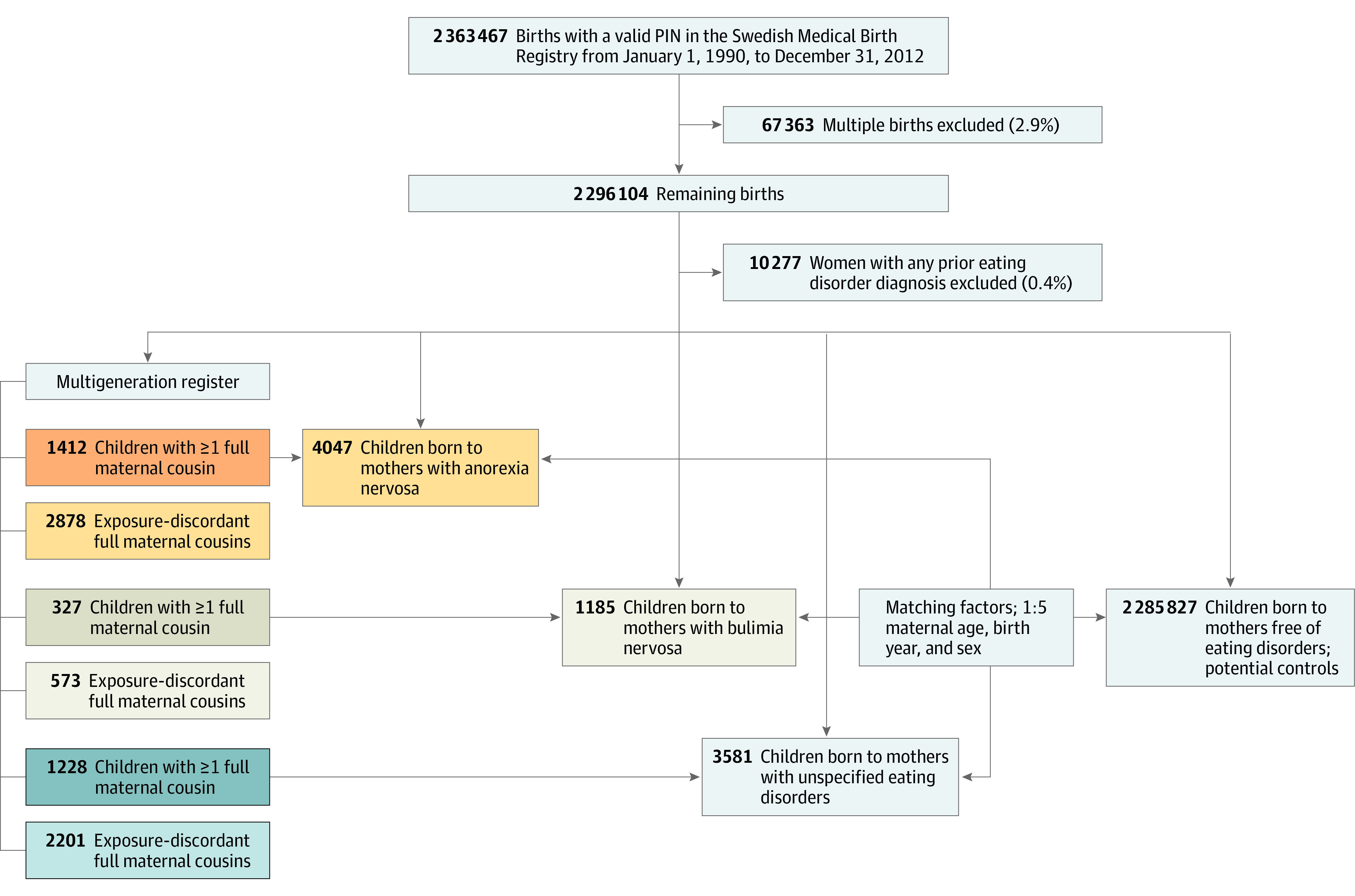
Flowchart of Study Population Generation PIN indicates personal identification number.

The study setting was the Swedish health care system, and data sources used are described in detail in the eMethods and eFigure 1 in the Supplement. We used the Swedish Medical Birth Registry and identified 2 296 074 of 2 363 437 singleton births (98.6%) with valid information on maternal and child personal identification numbers^16^ from January 1, 1990, to December 31, 2012, which made up the study base from which exposed children and matched unexposed comparator children were identified (Figure 1). Using the personal identification number, information from the Swedish Medical Birth Registry was linked with information from several national health registers to identify exposures, outcomes, and other covariates of interest. We obtained data from the National Patient Register, including information on inpatient and outpatient care, and the Cause of Death Register, which stores information on diseases or cause of death according to *International Classification of Diseases, Ninth Revision* (*ICD-9*), and *International Statistical Classification of Diseases and Related Health Problems, Tenth Revision* (*ICD-10*). We also retrieved information from the Prescribed Drug Register, including information on all dispensed prescriptions using Anatomical Therapeutic classifications (ATC), the Education Register, the Multigeneration Register, and the Total Population Register.

### Exposed Cohorts and Matched Comparators

Within the study base, 3 cohorts of exposed children were identified. Maternal eating disorder exposure was stratified into the subtypes anorexia nervosa, bulimia nervosa, and unspecified eating disorder and defined as (1) a maternal eating disorder diagnosis registered at least twice (anorexia nervosa, *ICD-9* 307B and *ICD-10* F50.0 and F50.1; bulimia nervosa, *ICD-10* F50.2 and F50.3; unspecified eating disorder, *ICD-9* 307F and *ICD-10* F50-9) in the Patient Register or (2) 1 main diagnosis in inpatient care before delivery date. Participants diagnosed before 9 years of age (and no registered diagnoses thereafter) were excluded to avoid misclassification of pediatric feeding disorders. Eating disorder status was further divided into ongoing disease, defined as at least 1 visit listing the specific diagnosis during the pregnancy or within 1 year before estimated conception, or previous eating disorder, defined as a last registered diagnosis more than 1 year before estimated conception.

All exposed children were matched with 5 comparator children, free of any maternal eating disorder exposure, based on maternal age at delivery, sex, and birth year. To adjust for unmeasured shared familial factors, we used the Multigeneration Register and identified a cluster of all exposed children with at least 1 exposure-discordant full maternal cousin (Figure 1).

### Outcomes and Follow-up

Based on the generally accepted minimal attained age for diagnosis of respective outcome, the follow-up started at different index ages depending on the specific outcome. Autism spectrum disorder (ASD) was defined as at least 2 registered diagnoses (*ICD-9* 299 and *ICD-10* F84.0, F84.1, F84.3, or F84.5) in the patient register during the follow-up period, and follow-up started at the index age of 1 year. Attention-deficit/hyperactivity disorder (ADHD) was defined as at least 2 registered diagnoses in the Patient Register (*ICD-9* 314 and *ICD-10* F90) and/or dispensed prescription of ADHD-specific pharmacotherapy (amphetamine [ATC N06BA01], dexamphetamine [ATC N06BA02], methylphenidate hydrochloride [ATC N06BA04], atomoxetine hydrochloride [ATC N06BA09], and lisdexamfetamine dimesylate [ATC N06BA12]) in the Prescribed Drug Register. The follow-up for ADHD started at the index age of 3 years. All children who died or migrated from Sweden before the index age were excluded from analyses. The follow-up started at the index age and ended at the point of outcome, December 31, 2017, death, or migration from Sweden. For the full maternal cousin cluster, the follow-up ended at point of outcome, death, migration from Sweden, or the minimal attained age (from index age to December 31, 2017) within the cluster.

### Covariates

Data on baseline pregnancy, delivery, and neonatal characteristics were obtained from the Swedish Medical Birth Registry and categorized as described in eTable 1 in the Supplement. Via the Multigeneration Register, we identified the personal identification number of the biological father for 98.1% to 99.0% of children. Data on maternal and paternal educational level were retrieved from the Educational Register as a proxy for socioeconomic status. For all mothers and fathers, a history of psychiatric (anxiety, depressive disorder, ADHD, or ASD), alcohol abuse, or substance abuse disorders was defined as a registered diagnosis in the Patient Register before the delivery date (*ICD-9* and *ICD-10* codes specified in eTable 2 in the Supplement).

### Statistical Analysis

Data were analyzed from August 31, 2020, to April 30, 2021. The exposed children of mothers with eating disorders were compared with the matched unexposed comparator children of mothers without eating disorders. We calculated crude incidence rates by dividing the number of respective outcome-events during follow-up with the corresponding person-time at risk presented as number of events per 1000 person-years. We used multivariable Cox proportional hazards regression models with robust standard variance estimates to obtain hazard ratios (HRs) as a measurement of the association between each maternal eating disorder subtype and offspring risk of the respective outcome. The proportional hazard assumption was assessed by inspecting cumulative incidence curves (eFigure 4 in the Supplement). The model was adjusted stepwise for confounders selected on the a priori hypothesized association with maternal eating disorder and neurodevelopmental disorder (eFigure 3 in the Supplement).^16^ Adjustments were made for matching factors, parental (maternal and paternal) educational level, paternal age at birth, maternal smoking status, and parental anxiety disorder, depressive disorder, ADHD, ASD, and alcohol or substance abuse disorder before the delivery date. Covariates with missingness were accounted for using complete case analysis. Stratum-specific HRs were calculated and presented for ongoing vs previous maternal eating disorder.

To investigate the potential effect of shared unmeasured genetic and environmental factors, we performed a stratified Cox proportional hazards regression model in the cluster of exposed children and their full maternal cousin comparators accounting for 12.5% of the genetic background. Only exposure-discordant full maternal cousin comparators were included in the analyses, and by design only outcome-discordant comparators contributed to the estimate.

Given the overlap of maternal eating disorder subtypes that, as expected, was most prominent for unspecified eating disorder (eFigure 2 in the Supplement), a sensitivity analysis was performed among children exposed to a maternal unspecified eating disorder without any overlap with anorexia nervosa and/or bulimia nervosa. We also repeated main analyses stratified by sex and by birth cohorts. Data were analyzed using SAS software, version 9.4 (SAS Institute Inc).

## Results

### Study Population and Baseline Characteristics

Figure 1 summarizes the study population generation and Table 1 summarizes the baseline characteristics of study participants (52 878 children). Swedish law does not allow data on race and ethnicity. The mean maternal (SD) age at delivery was 29.2 (5.1) years among mothers with anorexia nervosa and matched comparators, 29.6 (4.9) years among mothers with bulimia nervosa and matched comparators, and 28.9 (5.3) years among mothers with unspecified eating disorder and matched comparators. Among 4047 mothers with anorexia nervosa, 417 (10.3%) had ongoing disease during pregnancy, as did 803 of 3581 (22.4%) of mothers with unspecified eating disorder and 339 of 1185 (28.6%) of mothers with bulimia nervosa. Underweight (body mass index [calculated as weight in kilograms divided by height in meters squared], <18.5) was more frequent among mothers with anorexia nervosa (341 of 4047 [8.4%]) and unspecified eating disorder (201 of 3581 [5.6%]) compared with the matched comparator of group mothers (433 of 20 235 [2.1%] and 427 of 17 905 [2.4%], respectively). Children of mothers with eating disorders were more often firstborn (mothers with anorexia nervosa, 51.9%; mothers with unspecified eating disorder, 53.1%; and mothers with bulimia nervosa, 54.9%) compared with their matched comparators (range, 45.8%-47.9%). Maternal smoking, in particular before pregnancy, was more common among exposed children (range, 20.5%-26.2%) compared with their matched comparators (range, 17.1%-18.7%). Preterm birth was more common among exposed children (range, 6.3%-8.0%) compared with their matched unexposed comparator children (range, 4.7%-4.9%) (Table 1).

**Table 1.** Maternal Pregnancy, Delivery, and Neonatal Characteristics of Exposed Children of Mothers With Eating Disorder and Their Matched Comparators^a^

Characteristic	Maternal anorexia nervosa		Maternal bulimia nervosa		Maternal unspecified eating disorder	
	Exposed	Unexposed comparator	Exposed	Unexposed comparator	Exposed	Unexposed comparator
Ongoing disease^b^	417/4047 (10.3)	NA	339/1185 (28.6)	NA	803/3581 (22.4)	NA
Maternal age, y						
Mean (SD)	29.2 (5.1)	29.2 (5.1)	29.6 (4.9)	29.6 (4.9)	28.9 (5.3)	28.9 (5.3)
<20	88/4047 (2.2)	440/20 235 (2.2)	16/1185 (1.4)	80/5925 (1.3)	89/3581 (2.5)	445/17 905 (2.5)
20-25	937/4047 (23.2)	4685/20 235 (23.2)	234/1185 (19.7)	1170/5925 (19.7)	932/3581 (26.0)	4660/17 905 (26.0)
26-30	1362/4047 (33.7)	6810/20 235 (33.7)	440/1185 (37.1)	2200/5925 (37.1)	1197/3581 (33.4)	5985/17 905 (33.4)
31-35	1179/4047 (29.1)	5895/20 235 (29.1)	356/1185 (30.0)	1780/5925 (30.0)	947/3581 (26.4)	4735/17 905 (26.4)
>35	481/4047 (11.9)	2405/20 235 (11.9)	139/1175 (11.7)	695/5925 (11.7)	416/3581 (11.6)	2080/17 905 (11.6)
BMI						
Median (IQR)	21.2 (19.6-23.1)	23.5 (21.4-26.5)	22.9 (20.9-25.6)	23.7 (21.5-26.7)	22.2 (20.4-24.8)	23.5 (21.4-26.6)
<18.5	341/4047 (8.4)	433/20 235 (2.1)	41/1185 (3.5)	132/5925 (2.2)	201/3581 (5.6)	427/17 905 (2.4)
18.5-25.0	2715/4047 (67.1)	11 082/20 235 (54.8)	680/1185 (57.4)	3315/5925 (55.9)	2148/3581 (60.0)	9805/17 905 (54.8)
25.1-30.0	304/4047 (7.5)	4270/20 235 (21.1)	206/1185 (17.4)	1382/5925 (23.3)	497/3581 (13.9)	3833/17 905 (21.4)
>30.0	54/4047 (1.3)	2001/20 235 (9.9)	105/1185 (8.9)	636/5925 (10.7)	216/3581 (6.0)	1780/17 905 (9.9)
Missing	633/4047 (15.6)	2449/20 235 (12.1)	153/1185 (12.9)	460/5925 (7.8)	519/3581 (14.5)	2060/17 905 (11.5)
Parity						
Median (IQR)	1.7 (1.0-2.0)	1.8 (1.0-2.0)	1.0 (1.0-2.0)	2.0 (1.0-2.0)	1.7 (0.9)	1.8 (1.0)
1	2101/4046 (51.9)	9273/20 235 (45.8)	650/1185 (54.9)	2748/5925 (46.4)	1900/3581 (53.1)	8567/17 905 (47.8)
2	1296/4046 (32.0)	7385/20 235 (36.5)	372/1185 (31.4)	2178/5925 (36.8)	1123/3581 (31.3)	6415/17 905 (35.8)
3	433/4046 (10.7)	2604/20 235 (12.9)	113/1185 (9.5)	708/5925 (11.9)	401/3581 (11.2)	2095/17 905 (11.7)
>3	216/4046 (5.3)	973/20 235 (4.8)	50/1185 (4.2)	291/5925 (4.9)	157/3581 (4.4)	828/17 905 (4.6)
Smoking						
First antenatal visit	433/4047 (10.7)	1870/20 235 (9.2)	119/1185 (10.0)	429/5925 (7.2)	468/3581 (13.1)	1645/17 905 (9.2)
Missing	208/4047 (5.1)	994/20 235 (4.9)	48/1185 (4.1)	232/5925 (3.9)	152/3581 (4.2)	906/17 905 (5.1)
Before pregnancy	663/4047 (16.4)	2992/20 235 (14.8)	297/1185 (25.1)	969/5925 (16.4)	758/3581 (21.2)	2793/17 905 (15.6)
Missing	806/4047 (19.9)	3944/20 235 (19.5)	51/1185 (4.3)	262/5925 (4.4)	116/3581 (3.2)	2973/17 905 (16.6)
Mode of delivery						
Vaginal	3130/3996 (78.3)	15 458/19 980 (77.4)	880/1173 (75.0)	4557/5903 (77.2)	2664/3524 (75.6)	13 729/17 690 (77.6)
Assisted vaginal	272/3996 (6.8)	1520/19 980 (7.6)	93/1173 (7.9)	419/5903 (7.1)	245/3524 (7.0)	1369/17 690 (7.7)
Emergency cesarean delivery	251/3996 (6.3)	1482/19 980 (7.4)	85/1173 (7.2)	493/5903 (8.3)	243/3524 (6.9)	1353/17 690 (7.6)
Planned cesarean delivery	343/3996 (8.6)	1520/19 980 (7.6)	115/1173 (9.8)	434/5903 (7.3)	372/3524 (10.5)	1239/17 690 (7.0)
Preterm birth^c^						
All	324/4047 (8.0)	992/20 235 (4.9)	75/1185 (6.3)	282/5925 (4.7)	268/3581 (7.5)	853/17 905 (4.8)
Moderate	277/4047 (6.8)	866/20 235 (4.3)	64/1185 (5.4)	246/5925 (4.1)	222/3581 (6.2)	704/17 905 (3.9)
Very	32/4047 (0.8)	82/20 235 (0.4)	8/1185 (0.7)	23/5925 (0.4)	32/3581 (0.9)	104/17 905 (0.6)
Extreme	15/4047 (0.4)	44/20 235 (0.2)	3/1185 (0.3)	13/5925 (0.2)	14/3581 (0.4)	45/17 905 (0.3)
GW for GA^d^						
Appropriate	3809/4038 (94.3)	18 957/20 171 (94.0)	1113/1182 (94.2)	5597/5918 (94.6)	3353/3569 (93.9)	16 787/17 860 (94.0)
Small	158/4038 (3.9)	549/20 171 (2.7)	41/1182 (3.5)	149/5918 (2.5)	117/3569 (3.3)	528/17 860 (3.0)
Large	71/4038 (1.8)	665/20 171 (3.3)	28/1182 (2.4)	172/5918 (2.9)	99/3569 (2.8)	545/17 860 (3.1)
Microcephaly^e^	75/4047 (1.9)	287/20 235 (1.4)	26/1185 (2.2)	94/5925 (1.6)	76/3581 (2.1)	286/17 905 (1.6)
Missing	149/4047 (3.7)	679/20 235 (3.3)	41/1185 (3.5)	144/5925 (2.4)	116/3581 (3.2)	556/17 905 (3.1)
Apgar score <7 at 5 min	57/4047 (1.4)	213/20 235 (1.1)	15/1185 (1.3)	72/5925 (1.2)	57/3581 (1.6)	193/17 905 (1.1)
Missing	18/4047 (0.4)	121/20 235 (0.6)	7/1185 (0.6)	27/5925 (0.5)	37/3581 (1.0)	89/17 905 (0.5)

Abbreviations: BMI, body mass index (calculated as weight in kilograms divided by height in meters squared); GA, gestational age; GW, gestational weight; NA, not applicable.

^a^ Unless otherwise indicated, data are expressed as number/total number (%) of participants.

^b^ Diagnosis of the eating disorder during pregnancy or within 1 year before conception.

^c^ Preterm defined as before gestational week 37 plus 0 days; moderate, gestational week 32 plus 0 days to 36 plus 6 days; very, gestational week 28 plus 0 days to 31 plus 6 days; and extreme, before gestational week 28 plus 0 days.

^d^ Appropriately defined as GW of −2 to +2 SDs for GA; small, GW below −2 SDs for GA; and large, GW above +2 SDs for GA.

^e^ Defined as head circumference at birth below −2 SDs for GA.

### Parental Educational Level and Psychiatric Comorbidity Status

Parental educational level and psychiatric comorbidities are presented in Table 2. Except for a higher frequency of an educational level of greater than 12 years of schooling among mothers with anorexia nervosa (2176 of 4047 [53.8%]) vs comparators (9681 of 20 235 [47.8%]), there were no major differences in educational level among mothers and fathers of exposed children compared with those of nonexposed children. All groups of mothers with eating disorders had a higher co-occurrence of psychiatric comorbidities. Likewise, although with a smaller difference in absolute numbers, fathers of exposed children had a higher co-occurrence of most psychiatric comorbidities (Table 2).

**Table 2.** Parental Sociodemographic Factors and Psychiatric Comorbidities of Exposed Children of Mothers With Eating Disorders and Their Matched Comparators

Characteristics	Children’s group^a^					
	Maternal anorexia nervosa		Maternal bulimia nervosa		Maternal unspecified eating disorder	
	Exposed	Unexposed comparator	Exposed	Unexposed comparator	Exposed	Unexposed comparator
**Maternal**						
Educational level, y						
<9	312/4047 (7.7)	1799/20 235 (8.9)	94/1185 (7.9)	518/5925 (8.7)	396/3581 (11.1)	1702/17 905 (9.5)
9-12	1468/4047 (36.3)	8202/20 235 (40.5)	449/1185 (37.9)	2210/5925 (37.3)	1468/3581 (41.0)	7190/17 905 (40.2)
>12	2176/4047 (53.8)	9681/20 235 (47.8)	631/1185 (53.2)	3052/5925 (51.5)	1642/3581 (45.9)	8490/17 905 (47.4)
Missing	91/4047 (2.2)	553/20 235 (2.7)	11/1185 (0.9)	145/5925 (2.5)	75/3581 (2.1)	523/17 905 (2.9)
Psychiatric comorbidities^b^						
Depressive disorder	856/4047 (21.2)	534/20 235 (2.6)	357/1185 (30.1)	179/5925 (3.0)	1392/3581 (38.9)	492/17 905 (2.7)
Anxiety disorder	592/4047 (14.6)	420/20 235 (2.1)	554/1185 (46.8)	194/5925 (3.3)	996/3581 (27.8)	474/17 905 (2.6)
ADHD	63/4047 (1.6)	65/20 235 (0.3)	33/1185 (2.8)	19/5925 (0.3)	109/3581 (3.0)	50/17 905 (0.3)
ASD	27/4047 (0.7)	8/20 235 (0.04)	6/1185 (0.5)	7/5925 (0.1)	36/3581 (1.0)	12/17 905 (0.1)
Substance abuse	223/4047 (5.5)	105/20 235 (0.5)	126/1185 (10.6)	37/5925 (0.6)	344/3581 (9.6)	102/17 905 (0.6)
**Paternal** ^c^						
Age, mean (SD), y	32.8 (6.2)	32.6 (6.3)	32.8 (6.2)	33.3 (6.1)	32.2 (6.4)	32.6 (6.3)
Educational level, y						
<9	358/4000 (9.0)	2416/20 014 (12.1)	121/1166 (10.4)	612/5865 (10.4)	355/3511 (10.1)	2133/17 700 (12.1)
9-12	1947/4000 (48.7)	9595/20 014 (47.9)	590/1166 (50.6)	2818/5865 (48.0)	1811/3511 (51.6)	8623/17 700 (48.7)
>12	1579/4000 (39.5)	7275/20 014 (36.3)	430/1166 (36.9)	2257/5865 (38.5)	1231/3511 (35.1)	6326/17 700 (35.7)
Missing	116/4000 (2.9)	728/20 014 (3.6)	25/1166 (2.1)	178/5865 (3.0)	114/3511 (3.2)	618/17 700 (3.5)
Psychiatric comorbidities^b^						
Depressive disorder	96/4000 (2.4)	260/20 014 (1.3)	57/1166 (4.9)	106/5865 (1.8)	152/3511 (4.3)	230/17 700 (1.3)
Anxiety disorder	90/4000 (2.3)	223/20 014 (1.1)	36/1166 (3.1)	85/5865 (1.4)	116/3511 (3.3)	240/17 700 (1.4)
ADHD	30/4000 (0.8)	51/20 014 (0.3)	12/1166 (1.0)	24/5865 (0.4)	44/3511 (1.3)	86/17 700 (0.5)
ASD	3/4000 (0.1)	6/20 014 (0.03)	3/1166 (0.3)	1/5865 (0.02)	4/3511 (0.1)	4/17 700 (0.02)
Substance abuse	65/4000 (1.6)	120/20 014 (0.6)	27/1166 (2.3)	46/5865 (0.8)	82/3511 (2.3)	130/17 700 (0.7)

Abbreviations: ADHD, attention-deficit/hyperactivity disorder; ASD autism spectrum disorder.

^a^ Unless otherwise indicated, data are expressed as number/total number (%) of children.

^b^ Registered diagnosis in the patient register before delivery date.

^c^ Includes number/total number (%) of fathers with valid information from the personal identification number.

### The Offspring Risk of ADHD

**Figure 2.**
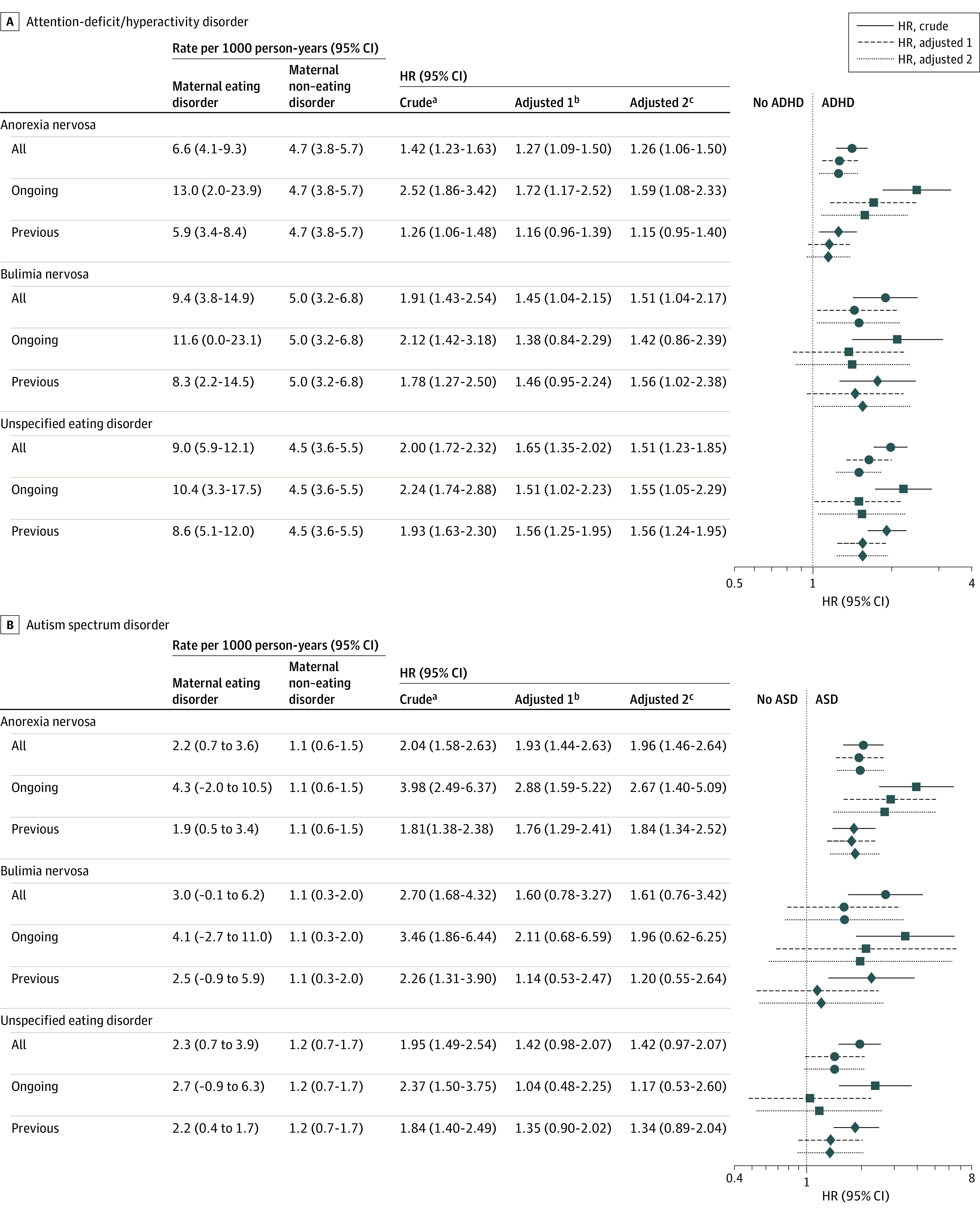
Forest Plot of the Hazard Ratios (HRs) of Attention-Deficit/Hyperactivity Disorder (ADHD) and Autism Spectrum Disorder (ASD) Comparisons are between children of mothers with eating disorders vs mothers without eating disorders. Circles indicate all children; squares, children with ongoing disease; diamonds, children with previous disease. Parent psychiatric comorbidities adjusted for included anxiety disorder, depressive disorder, ADHD, and ASD. ^a^Adjusted for maternal age at birth, sex, and birth year. ^b^Adjusted for maternal smoking status, parity, maternal educational level, maternal psychiatric comorbidities, and maternal alcohol or substance use disorder in addition to factors adjusted for in crude model. ^c^Adjusted for paternal educational level, psychiatric comorbidities, and alcohol or substance use disorder in addition to factors adjusted for in the first adjusted model.

During a mean (SD) follow-up time of 9.7 (5.5) years, there was an overall 40% increased risk of ADHD (crude HR, 1.42 [95% CI, 1.23-1.63]) among children of mothers with anorexia nervosa. The risk decreased to 27% (fully adjusted HR, 1.26 [95% CI, 1.06-1.50]) after additional adjusting for parental educational level and psychiatric comorbidities. Stratification revealed a higher risk among children to mothers with ongoing anorexia nervosa during pregnancy (crude HR, 2.52 [95% CI, 1.86-3.42]; fully adjusted HR, 1.59 [95% CI, 1.08-2.33]) compared with children of mothers with previous anorexia nervosa for whom adjusted HRs were no longer significantly increased (fully adjusted HR, 1.15 [95% CI, 0.95-1.40]). Children of mothers with bulimia nervosa and an unspecified eating disorder were also at increased risk of ADHD during a mean (SD) follow-up of 6.8 (3.0) years and 8.7 (5.3) years, respectively (crude HR among children exposed to maternal bulimia nervosa, 1.91 [95% CI, 1.43-3.18]; crude HR among children exposed to maternal unspecified eating disorder, 2.00 [95% CI, 1.72-2.32]). Hazard ratios remained significantly increased after adjusting for parental educational level and psychiatric comorbidities (fully adjusted HRs, 1.51 [95% CI, 1.04-2.17] and 1.51 [95% CI, 1.23-1.85], respectively). Stratification of bulimia nervosa into ongoing vs previous disease did not reveal any significant difference in crude estimate, whereas adjusted estimates were attenuated and no longer significant. Stratification of unspecified eating disorder into ongoing vs previous disease did not reveal any significant differences in estimates (Figure 2A).

### The Offspring ASD Risk

The risk of ASD was increased among children born to mothers with anorexia nervosa (crude HR, 2.04 [95% CI, 1.58-2.63]) and remained similarly increased after adjusting for parental educational level and psychiatric comorbidities (fully adjusted HR, 1.96 [95% CI, 1.46-2.64]) during a mean (SD) follow-up of 10.8 (5.6) years. Children of mothers with ongoing anorexia nervosa had a 4-fold increased risk of ASD (crude HR, 3.98 [95% CI, 2.49-6.37]; fully adjusted HR, 2.67 [95% CI, 1.40-5.09]) compared with an 80% increased risk for children of mothers with previous anorexia nervosa (crude HR, 1.81 [95% CI, 1.38-2.38]; fully adjusted HR, 1.84 [95% CI, 1.34-2.52]). The crude HRs for ASD among children of mothers with bulimia nervosa (HR, 2.70 [95% CI, 1.68-4.32]) and an unspecified eating disorder (HR, 1.95 [95% CI, 1.49-2.54]) were similar to those of children born to mothers with anorexia nervosa, but estimates of the stratified fully adjusted model did not remain significantly increased (Figure 2B).

### Within-Family and Sensitivity Analyses

Complete baseline characteristics of the exposed children, by maternal eating disorder subtype, and their full maternal cousins are presented in eTables 3 and 4 in the Supplement. The results based on the within-family analysis (Table 3) were similar to those of the main analysis for the maternal anorexia nervosa cohort (fully adjusted HR for ADHD, 1.85 [95% CI, 1.06-3.22]; fully adjusted HR for ASD, 1.90 [95% CI, 0.47-7.65]), whereas estimates for the maternal unspecified eating disorder cohort diminished and were no longer significant (fully adjusted HR for ADHD, 1.17 [95% CI, 0.72-1.91]; fully adjusted HR for ASD, 0.79 [95% CI, 0.21-2.95]). For the maternal bulimia nervosa cohort, the risk of ADHD was more pronounced compared with the main analysis (fully adjusted HR, 30.92 [95% CI, 2.56-373.19]), and the analysis for ASD could not be performed owing to the small sample size.

**Table 3.** Within-Family Analysis (Full Maternal Cousin Clusters) of the Relative Risk of Neuropsychiatric Diseases Among Children of Mothers With Eating Disorders

Maternal diagnosis	ADHD				ASD			
	No of cases^a^	HR (95% CI)			No of cases^a^	HR (95% CI)		
		Crude^b^	Partially adjusted^c^	Fully adjusted^d^		Crude^b^	Partially adjusted^c^	Fully adjusted^d^
Anorexia nervosa	1400	1.59 (1.03-2.45)	2.06 (1.22-3.48)	1.85 (1.06-3.22)	1410	2.25 (1.08-4.69)	3.28 (1.12-9.65)	1.90 (0.47-7.65)
Bulimia nervosa	351	7.32 (2.27-23.57)	33.33 (2.86-389.07)	30.92 (2.56-373.19)	354	3.16 (0.18-52.21)	NA	NA
Unspecified eating disorder	1110	1.11 (0.72-1.70)	1.17 (0.73-1.87)	1.17 (0.72-1.91)	1125	1.11 (0.39-3.13)	0.82 (0.22-2.98)	0.79 (0.21-2.95)

Abbreviations: ADHD, attention-deficit hyperactivity disorder; ASD, autism spectrum disorder; HR, hazard ratio; NA, not applicable.

^a^ Indicates the number of families with full maternal cousins discordant for both maternal eating disorder exposure and respective neuropsychiatric disease outcome (contributing to estimate by design, but all exposure-discordant families included in analysis).

^b^ Adjusted for maternal age at delivery, sex, and birth year.

^c^ Adjusted for maternal psychiatric comorbidities (anxiety disorder, depressive disorder, ADHD, and ASD) and alcohol or substance use disorder in addition to factors adjusted for in crude model.

^d^ Adjusted for paternal psychiatric comorbidities (anxiety disorder, depressive disorder, ADHD, and ASD) and alcohol or substance use disorder in addition to factors adjusted for in the partially adjusted model.

Analysis of unspecified eating disorder exposure without overlapping specific eating disorder subtypes was similar to the main analysis (eTable 5 in the Supplement). There were no distinct patterns or significant differences in analysis stratified by child sex (eTable 6 in the Supplement) or birth cohort period (eTable 7 in the Supplement).

## Discussion

In this population-based prospective cohort study, we observed an increased risk of ADHD and ASD among children of mothers with eating disorders, regardless of subtype, compared with children of mothers free of eating disorder diagnoses. Furthermore, we observed a tendency to a higher risk among children of mothers with ongoing eating disorders that was most prominent among children to mothers with anorexia nervosa.

Previous studies^14,15^ have observed poorer neurobehavioral, language, and motor development (which could be indicative of neuropsychiatric disorders) in children of mothers with self-reported eating disorders, whereas we could associate maternal eating disorder exposure with neuropsychiatric diseases in an unselected study population. In contrast to previous studies, we could also provide information on and adjust for many potential confounders, as well as adjust for familial confounding. In general, the observed association could not be fully explained by parental psychiatric comorbidity status.

Stratification into previous vs ongoing eating disorder revealed a tendency toward a higher risk among children of mothers with ongoing eating disorders that, to the best of our knowledge, is a novel finding. Although our results do not provide information on causality, some assumptions can be generated. The risk of ADHD and ASD was twice as high (2.5-fold and 4.0-fold, respectively) among children of mothers with ongoing anorexia nervosa compared with children of mothers with previous anorexia nervosa. Hypothetically, these results are suggestive of an actual effect of disease-specific factors mediating the association between a maternal eating disorder and childhood neuropsychiatric disorder. For example, ongoing anorexia nervosa is associated with several abnormal metabolic^17,18^ and endocrine biomarkers.^17^ Epigenetic changes have repeatedly been suggested a potential mediator between maternal exposures and childhood outcomes,^19^ and specific nutritional deficiencies have been linked to DNA hypomethylation.^20^ In fact, 1 study^21^ found lower levels of cord blood DNA methylation, specifically in genes relevant for neuronal development, in offspring of mothers with active eating disorder.

Interestingly, the risk of ADHD and ASD was also increased in children of mothers with previous eating disorder. Whether mothers with previous eating disorder were in complete disease remission during pregnancy is not known. Given the substantial number of women with eating disorders for whom the disease course is chronic^4,22^ and there is potential risk of relapse during pregnancy,^5,6^ there might be women with disease relapse and/or residual eating disorder symptoms during pregnancy within the group who would be defined as having a previous eating disorder. Moreover, eating disorders are associated with an increased co-occurrence of other psychiatric conditions,^23^ which also was observed in the present study. Several positive genetic correlations between eating disorders and other psychiatric diseases, including ADHD and eating disorders other than anorexia nervosa,^18^ have been described. Hence, intergenerational transmission of a genetic risk of specific psychiatric phenotypes is another potential rationale for the association between maternal eating disorders and childhood neuropsychiatric diseases. To control for genetic factors, we adjusted for parental psychiatric comorbidities, which attenuated the estimates of the main analysis slightly, but most remained significantly increased. Interestingly, maternal anorexia nervosa exposure estimates were generally attenuated to a lesser extent compared with estimates of other eating disorder exposures. Hypothetically, this difference indicates that genetic transmission is associated with the risk of ADHD and ASD in children of mothers with anorexia nervosa to a lesser extent compared with children of mothers with bulimia nervosa or an unspecified eating disorder. In addition, we performed an analysis based on exposed children compared with their full maternal cousins accounting for 12.5% of their genetic risk, in which the estimates of children of mothers with anorexia nervosa did not change significantly compared with the main analysis. The results from the family analysis supports the hypothesis of a low genetic transmission effect on the association between maternal anorexia nervosa exposure and offspring neuropsychiatric risk. Estimates of children of mothers with bulimia nervosa remained significantly increased for ADHD, but with poor precision owing to limited number of participants and outcomes. Clearly, we could only adjust for a minor part of the genetic risk and can therefore not conclude that the observed association is not mediated via familial confounding, but presumably the association would have been attenuated if this was the case.

### Strengths and Limitations

Our study has several major strengths, including the population-based approach and use of nationwide registers with prospectively collected data, precluding recall bias, and an established high validity of most diagnoses.^19^ However, our results should be interpreted within the context of the study’s limitations. Although the positive predictive values of most diagnoses are high in data sources from which exposure status was derived,^24^ the validity of eating disorders has not been assessed. Moreover, eating disorders are still stigmatized diseases, meaning that there might still be unrecorded cases and a risk of selecting cases with more severe disease. Furthermore, the outpatient part of the patient register was initiated in 2001, which is why there may be a difference in the clinical phenotype (ie, severity) of eating disorders before vs after this time. We attempted to address this (and the increasing trend of both eating disorders and neuropsychiatric diagnoses) by matching on birth year. The more frequent contact with health care in combination with a potential tendency to increased awareness of developmental deviations in children among patients with ongoing diseases such as eating disorders might result in a differential risk of disease detection compared with healthy individuals (surveillance bias). In addition, although we adjusted for a large number of predefined confounders, there might still be residual confounding, and likewise we could only adjust for a small part of unmeasured familial confounding. Finally, despite being one of the largest studies to date (to our knowledge), we acknowledge that numbers and precision were sometimes poor in stratified and adjusted analyses. The relatively short median follow-up time of 9 to 13 years of age does not introduce a bias per se but may impose difficulties generalizing the results to older groups.

## Conclusions

In this prospective nationwide cohort study, we found an association between maternal eating disorders and neuropsychiatric diseases in children that could not entirely be explained by parental comorbidities or familial confounding. The risk of neuropsychiatric diseases was highest among children of mothers with ongoing eating disorders during pregnancy. In addition to stressing the need of future research addressing factors mediating this association, our results also implicate the importance of clinical awareness and intensified support to women with eating disorders and their children.

## Notes

Supplement. **eMethods.** Study Setting and Data Linkage **eFigure 1.** Overview of Study Data Linkage and Covariates Obtained From Respective Data Source **eFigure 2.** Illustration of Overlap Between Exposed Cohorts **eFigure 3.** Hypothesized Directed Acyclic Graph of Maternal Eating Disorder and Neurodevelopmental Disorder in Children **eFigure 4.** Cumulative Incidence of Neuropsychiatric Outcomes Among Children of Mothers With Eating Disorder and Their Controls **eTable 1.** Definition of Pregnancy, Delivery, and Neonatal Characteristics Obtained From the Medical Birth Registry **eTable 2.** *International Classification of Disease* Codes Used to Identify Parental Comorbidities **eTable 3.** Maternal Pregnancy, Delivery, and Neonatal Characteristics of Exposed Children of Mothers With Eating Disorder and Their Full Maternal Cousins **eTable 4.** Parental Sociodemographic Factors and Psychiatric Comorbidities of Exposed Children of Mothers With Eating Disorder and Their Full Maternal Cousins **eTable 5.** Relative Risk of Neurodevelopmental Disorders Among Children of Mothers With Unspecified Eating Disorder Without Overlapping Diagnoses **eTable 6.** Relative Risk of Neurodevelopmental Disorders Among Children of Mothers With Eating Disorder Stratified by Sex **eTable 7.** Relative Risk of Neurodevelopmental Disorders Among Children of Mothers With Eating Disorder Stratified by Birth Cohort
eReferences
