# Supplementary material for: Analysis of Neurodevelopmental Disorders in Offspring of Mothers With Eating Disorders in Sweden
Source: JAMA Netw Open. 2022 Jan 18;5(1):e2143947. doi: 10.1001/jamanetworkopen.2021.43947 (PMC8767445; doi:10.1001/jamanetworkopen.2021.43947)
Supplement: Supplement. — eMethods. Study Setting and Data Linkage eFigure 1. Overview of Study Data Linkage and Covariates Obtained From Respective Data Source eFigure 2. Illustration of Overlap Between Exposed Cohorts eFigure 3. Hypothesized Directed Acyclic Graph of Maternal Eating Disorder and Neurodevelopmental Disorder in Children eFigure 4. Cumulative Incidence of Neuropsychiatric Outcomes Among Children of Mothers With Eating Disorder and Their Controls eTable 1. Definition of Pregnancy, Delivery, and Neonatal Characteristics Obtained From the Medical Birth Registry eTable 2. International Classification of Disease Codes Used to Identify Parental Comorbidities eTable 3. Maternal Pregnancy, Delivery, and Neonatal Characteristics of Exposed Children of Mothers With Eating Disorder and Their Full Maternal Cousins eTable 4. Parental Sociodemographic Factors and Psychiatric Comorbidities of Exposed Children of Mothers With Eating Disorder and Their Full Maternal Cousins eTable 5. Relative Risk of Neurodevelopmental Disorders Among Children of Mothers With Unspecified Eating Disorder Without Overlapping Diagnoses eTable 6. Relative Risk of Neurodevelopmental Disorders Among Children of Mothers With Eating Disorder Stratified by Sex eTable 7. Relative Risk of Neurodevelopmental Disorders Among Children of Mothers With Eating Disorder Stratified by Birth Cohort eReferences [file jamanetwopen-e2143947-s001.pdf]

Supplementary Online Content

Mantel Ä, Örtqvist AK, Hirschberg AL, Stephansson O. Analysis of neurodevelopmental disorders in offspring of mothers with eating disorders in Sweden. *JAMA Netw Open*. 2022;5(1):e2143947. doi:10.1001/jamanetworkopen.2021.43947

eMethods. Study Setting and Data Linkage

eFigure 1. Overview of Study Data Linkage and Covariates Obtained From Respective Data Source

eFigure 2. Illustration of Overlap Between Exposed Cohorts

eFigure 3. Hypothesized Directed Acyclic Graph of Maternal Eating Disorder and Neurodevelopmental Disorder in Children

eFigure 4. Cumulative Incidence of Neuropsychiatric Outcomes Among Children of Mothers With Eating Disorder and Their Controls

eTable 1. Definition of Pregnancy, Delivery, and Neonatal Characteristics Obtained From the Medical Birth Registry

eTable 2. *International Classification of Disease* Codes Used to Identify Parental Comorbidities

eTable 3. Maternal Pregnancy, Delivery, and Neonatal Characteristics of Exposed Children of Mothers With Eating Disorder and Their Full Maternal Cousins

eTable 4. Parental Sociodemographic Factors and Psychiatric Comorbidities of Exposed Children of Mothers With Eating Disorder and Their Full Maternal Cousins

eTable 5. Relative Risk of Neurodevelopmental Disorders Among Children of Mothers With Unspecified Eating Disorder Without Overlapping Diagnoses

eTable 6. Relative Risk of Neurodevelopmental Disorders Among Children of Mothers With Eating Disorder Stratified by Sex

eTable 7. Relative Risk of Neurodevelopmental Disorders Among Children of Mothers With Eating Disorder Stratified by Birth Cohort

eReferences

This supplementary material has been provided by the authors to give readers additional information about their work.

© 2022 Mantel Ä et al. *JAMA Network Open*.

eMethods. Study Setting and Data Linkage

Study setting

The Swedish health care system is tax-funded and includes free of charge maternal health care. Virtually all pregnant women attend the antenatal program and the frequency of antenatal visits and individual need of extra health resources is determined during the first antenatal visit, in the end of the first trimester, based on patient history and disease-status. Patients with uncomplicated eating disorder are seen in primary health care, whereas patients with more severe eating disorders are treated by psychiatrists (Adult or pediatric depending on age). There are specialized eating disorder clinics in all regions. Neuropsychiatric disorders are diagnosed and treated by psychiatrists.

Data linkage and nationwide Swedish registers

The Swedish personal identification number (PIN), which is unique for the individual, consists of the a six-digit birthdate followed by a four-digit identification number. The PIN is assigned by the Swedish tax agency to all inhabitants at birth or immigrants intending to stay ≥ 1 year. (1) In this study we used the PIN and linked several nationwide registers to obtain information on exposures, outcomes and covariates of interest.

Demographic registers are kept by Statistics Sweden and includes the following registers used in this study:

i) The total population register
ii) The longitudinal integration database for health insurance and labor market studies (LISA)
iii) The cause of death register
iv) The multigeneration register

Additionally, the national health registers are kept by the National Board of Health and Welfare (NBWH) and includes the following registers, which were also used in this study:

v) The medical birth register
vi) The national patient register
vii) The prescribed drug register which were also used in the study.

The total population register contains demographic information, including information on migrations. Information on migration during the follow-up period was obtained from the total population register. Parental educational level was obtained from LISA, which includes yearly updated information on educational level on all individuals above 16 years of age. Information on deaths was retrieved from the cause of death register, which since the 1960s contains date of deaths and supposed cause of death coded according to the International classification of diseases (ICD), to identify all deaths (competing events) during the follow-up period. The biologic fathers and the full maternal cousins were identified using the multi-generation register. The multigeneration register collects parental information on index persons and includes virtually complete parental information for contemporary generations.

The medical birth register contains information on more than 98% of all births in Sweden since 1973. Information is collected prospectively throughout the pregnancy using standardized records from antenatal, delivery and neonatal care. The medical birth register was used to identify a study base and to retrieve information on baseline pregnancy, delivery and neonatal characteristics which are specified in eTable 1. The Swedish national patient register includes information on inpatient care since 1964 (full coverage since 1987) and outpatient care since 2001. Main and secondary diagnoses are coded according to the ICD-system (ninth revision between 1987 and 1997 and tenth revision after 1998) by the assessing or discharging physician. The patient register was used to retrieve information on exposure (maternal eating disorder), outcomes (ADHD and ASD) and parental comorbidities (ICD-codes used in supplementary eTable x). Validity of most diagnoses, including psychiatric disorders, have proven high.(2) The prescribed drug register contains information on dispensed prescribed drugs from Swedish pharmacies, coded accorded to anatomical therapeutic chemicals (ATC) codes, since July 2005 and was used to retrieve data on ADHD-drugs as a proxy for outcome.

© 2022 Mantel Ä et al. *JAMA Network Open*.

eFigure 1. Overview of Study Data Linkage and Covariates Obtained From Respective Data Source

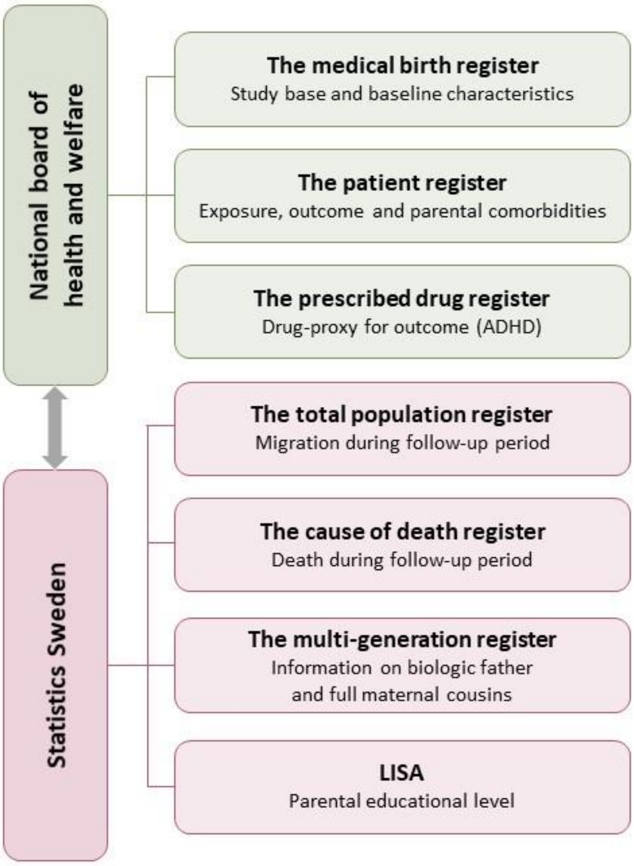

© 2022 Mantel Ä et al. *JAMA Network Open*.

eFigure 2. Illustration of Overlap Between Exposed Cohorts

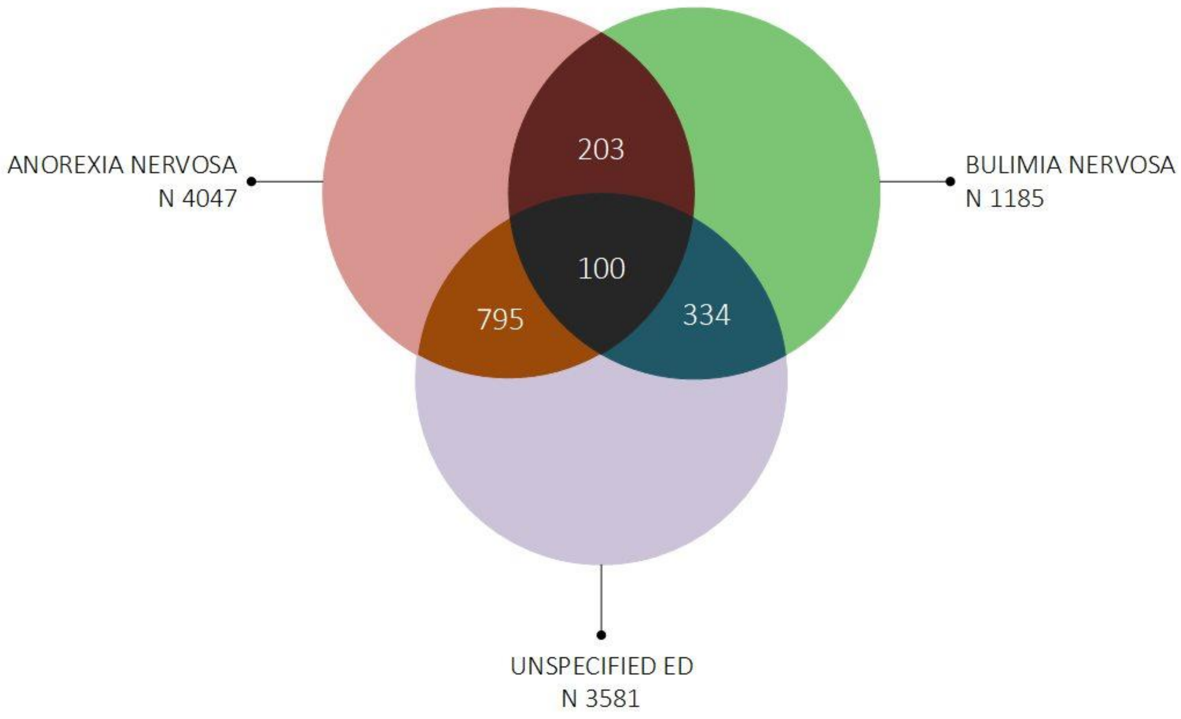

Figure illustrates the overlap between maternal eating disorders. In total, 100 mothers had a registered diagnosis of all eating disorder subtypes, 203 had been diagnosed with anorexia nervosa and bulimia nervosa, 795 had been diagnosed with anorexia nervosa and unspecified eating disorder and 334 had been diagnosed with bulimia nervosa and unspecified eating disorder.

ED, Eating disorder

© 2022 Mantel Ä et al. *JAMA Network Open*.

eFigure 3. Hypothesized Directed Acyclic Graph of Maternal Eating Disorder and Neurodevelopmental Disorder in Children

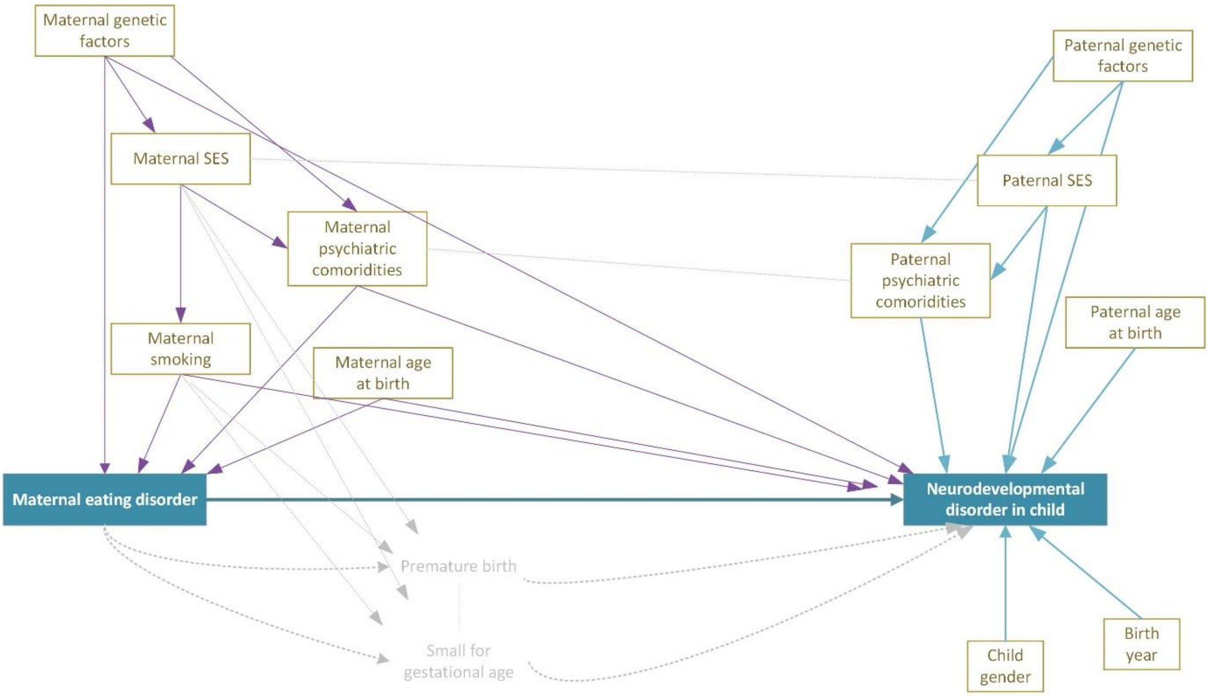

The directed acyclic graphs (DAG) shows the hypothesized direct effect of maternal eating disorder on neurodevelopmental disorders in the children, confounded by parental genetic factors, socioeconomic status (SES), demographics, psychiatric comorbidities and maternal smoking status. Additionally, the DAG also shows the potential mediated effect of premature birth and/or fetal growth.

© 2022 Mantel Ä et al. *JAMA Network Open*.

eFigure 4. Cumulative Incidence of Neuropsychiatric Outcomes Among Children of Mothers With Eating Disorder and Their Controls

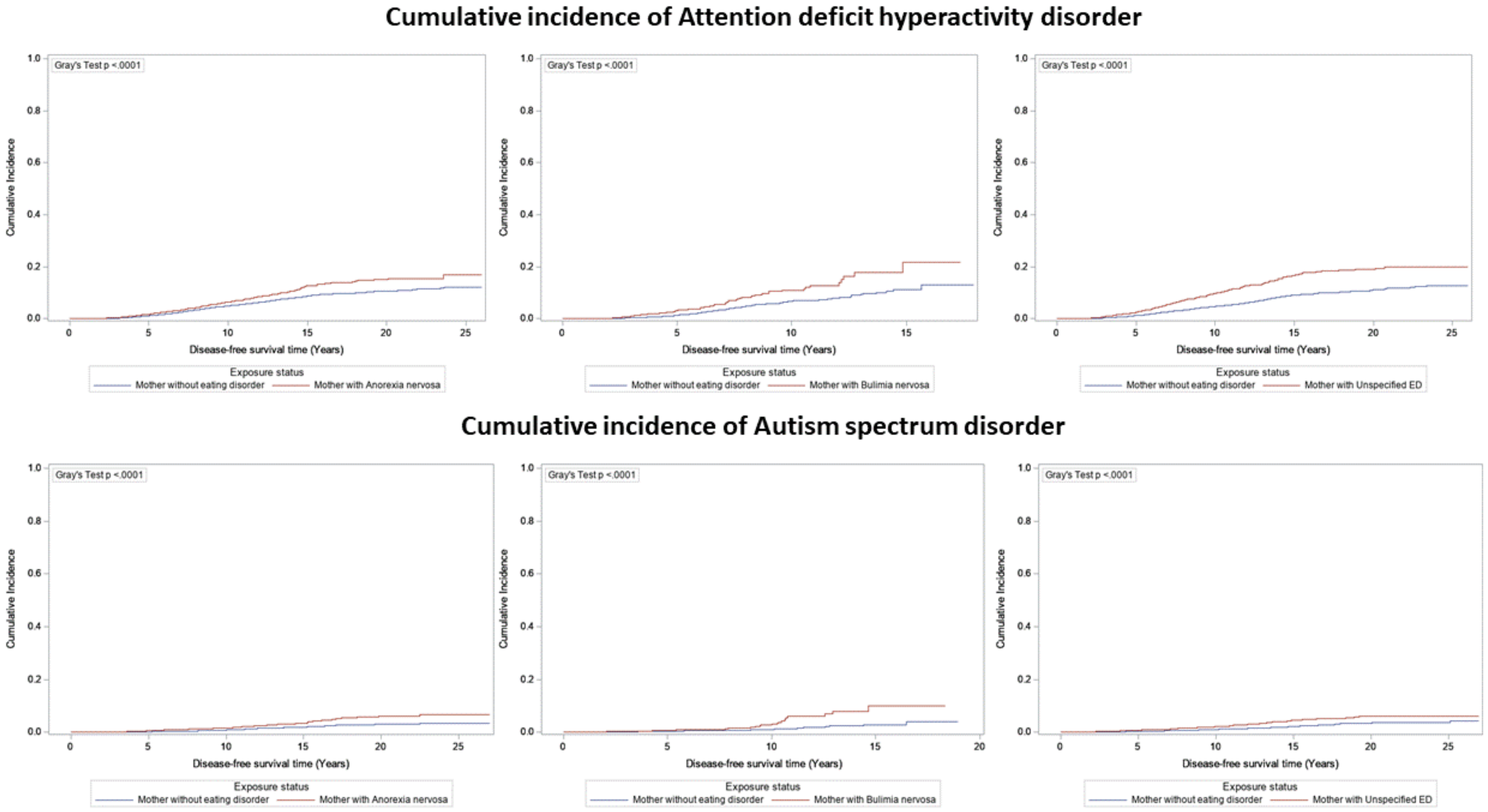

© 2022 Mantel Ä et al. *JAMA Network Open*.

eTable 1. Definition of Pregnancy, Delivery, and Neonatal Characteristics Obtained From the Medical Birth Registry

Variable	Definition and categorization
Maternal age	Mothers' age (in years) at delivery date<br>Categorized into age groups (<20 years of age, between 20 and 25 years of age, between 26 and 30 years of age, between 31 and 35 years of age and >35 years of age)
Paternal age	Fathers' age at delivery date
Body mass index (BMI)	Weight in kilograms/height in meters^2<br>Based on early pregnancy weight at first antenatal visit and self-reported height.<br>Categorized into WHOs nutritional status categories<br><18.5 (underweight)<br>18.5-24.9 (Normal weight)<br>25.0-29.9 (Overweight)<br>>30.0 (Obesity)
Parity	Including present delivery<br>Categorized into 1,2,3 and >3
Smoking status	Self-reported information on smoking before pregnancy and ongoing smoking at the first antenatal visits (end of first trimester)
Mode of delivery	As registered in the delivery medical record
Gestational weight for gestational age	Birth weight measured at birth<br>Swedish sex-specific estimated fetal growth curves were used to categorize gestational weight into:<br>Appropriate for gestational age (AGA) defined as gestational weight between two standard deviations below and 2 standard deviations above the mean birth weight for gestational their gestational age<br>Small for gestational age (SGA) defined as gestational weight less than 2 standard deviations below the mean birth weight for their gestational age<br>Large for gestational age (LGA) defined as gestational weight more than 2 standard deviations above the mean birth weight for gestational age
Microcephaly	Based on head circumference measured at birth<br>Microcephaly defined as a head circumference less than two standard deviations below the sex-specific mean for gestational age

© 2022 Mantel Ä et al. *JAMA Network Open*.

eTable 2. ***International Classification of Disease*** **Codes Used to Identify Parental Comorbidities**

Comorbidity	ICD Revision 9	ICD Revision 10
Depressive disorder	300E, 311	F32-F34
Anxiety disorder	300A/C	F40-F41
ADHD	314	F90
ASD	299A/B/X	F84.0/1/3/5
Alcohol and substance abuse disorder	303-304, 305A/X	F10-F16, F18-F19
ADHD, Attention-deficit hyperactivity disorder; ASD, Autism spectrum disorder; ICD, international classification of disease		

© 2022 Mantel Ä et al. *JAMA Network Open*.

eTable 3. Maternal Pregnancy, Delivery, and Neonatal Characteristics of Exposed Children of Mothers With Eating Disorder and Their Full Maternal Cousins

Characteristics	Children exposed to maternal Anorexia Nervosa N 1412	Exposure discordant full maternal cousins N 2878	Children exposed to maternal Bulimia Nervosa N 354	Exposure discordant Full maternal cousins N 695	Children exposed to maternal Unspecified ED N 1128	Exposure discordant full maternal cousins N 2201
Preterm birth†						
All – no. (%)	113 (8.0)	127 (4.4)	16 (4,5)	29 (4.2)	71 (6.3)	90 (4.1)
Moderate – no. (%)	98 (6.9)	119 (4.1)	13 (3.7)	27 (3.9)	55 (4.9)	71 (3.2)
Very – no. (%)	12 (0.9)	4 (0.1)	2 (0.6)	2 (0.3)	12 (1.1)	16 (0.7)
Extreme – no. (%)	3 (0.2)	4 (0.1)	1 (0.3)	0	4 (0.4)	3 (0.1)
Gestational weight for gestational age‡						
Appropriate – no (%)	1336 (94.8)	2745 (95.8)	330 (93.5)	656 (94.4)	1054 (93.8)	2033 (92.7)
Small – no. (%)	54 (3.8)	50 (1.8)	12 (3.4)	13 (1.9)	40 (3.6)	67 (3.1)
Large – no. (%)	20 (1.4)	69 (2.4)	11 (3.1)	26 (3.7)	30 (2.7)	93 (4.2)
Microcephaly – no. (%)§	27 (2.0)	30 (1.1)	5 (1.5)	7 (1.0)	23 (2.1)	33 (1.6)
Missing	56 (4.0)	119 (4.1)	10 (2.8)	25 (3.6)	34 (3.0)	79 (3.6)
Apgar < 7 at 5 minutes	13 (0.9)	33 (1.2)	3 (0.9)	4 (0.6)	16 (1.4)	10 (0.5)
Missing	5 (0.4)	26 (0.9)	2 (0.6)	5 (0.7)	13 (1.2)	18 (0.8)

ED, Eating disorder; BMI, Body Mass Index

†Preterm birth defined as < gestational week 37+0, Moderate preterm birth between gestational week 32+0 and 36+6, Very preterm birth between gestational week 28+0 and 31+6 and Extreme preterm birth < gestational week 28+0. ‡Appropriate for gestational age defined as gestational weight between -2 and +2 standard deviations for gestational age Small for gestational age defined as gestational weight below -2 standard deviations and large for gestational age defined as gestational weight above +2 standard deviations for gestational age.

§Microcephaly defined as head circumference at birth below -2 standard deviation for gestational age.

© 2022 Mantel Ä et al. *JAMA Network Open*.

eTable 3. ***Continued***

Characteristics	Children exposed to maternal Anorexia Nervosa N 1412	Exposure discordant full maternal cousins N 2878	Children exposed to maternal Bulimia Nervosa N 354	Exposure discordant full maternal cousins N 695	Children exposed to maternal Unspecified ED N 1128	Exposure discordant Full maternal cousins N 2201
Maternal age						
Mean yr ± SD	29.4 ± 5.0	28.6 ± 4.8	29.9 ± 5.1	28.0 ± 4.8	29.4 ± 5.0	28.2 ± 5.0
<20 yr – no. (%)	27 (1.9)	70 (2.4)	8 (2.3)	24 (3.5)	16 (1.4)	72 (3.3)
20-25 yr – no. (%)	297 (21.0)	716 (24.9)	59 (16.7)	189 (27.2)	245 (21.7)	619 (28.1)
26-30 yr – no. (%)	508 (36.0)	1104 (38.4)	130 (36.7)	276 (39.7)	411 (36.4)	778 (35.4)
31-35 yr – no. (%)	419 (29.7)	757 (26.3)	53 (15.0)	47 (6.8)	318 (28.2)	569 (25.9)
>35 yr – no. (%)	161 (11.4)	231 (8.0)			138 (12.2)	163 (7.4)
BMI						
Mean ± SD	21.5 ± 2.9	23.7 ± 4.3	23.4 (21.5-26.0)	23.7 (21.3-26.9)	22.1 (20.3-24.6)	23.5 (21.2-26.7)
<18.5 – no. (%)	122 (8.6)	81 (2.8)	7 (2.0)	21 (3.0)	64 (5.6)	67 (3.0)
18.5-25.0 – no. (%)	977 (69.2)	1680 (58.4)	201 (56.8)	340 (48.9)	680 (60.3)	1158 (52.6)
25.1-30.0 – no. (%)	95 (6.7)	446 (15.5)	71 (20.1)	169 (24.3)	149 (13.2)	434 (19.7)
>30.0 – no. (%)	14 (1.0)	204 (7.1)	34 (9.6)	74 (10.7)	66 (5.9)	216 (9.8)
Missing	204 (14.5)	467 (16.2)	41 (11.6)	91 (13.1)	169 (15.0)	326 (14.8)
Parity						
Median (IQR)	1.8 ± 1.1	1.8 ± 1.0	1.7 ± 0.9	1.8 ± 1.1	1.8 ± 1.0	1.8 ± 0.9
1 – no. (%)	709 (50.2)	1295 (45.0)	186 (52.5)	339 (48.8)	553 (49.0)	1037 (47.1)
2 – no. (%)	464 (32.9)	1029 (35.8)	115 (32.5)	251 (36.1)	365 (32.4)	761 (34.6)
3 – no. (%)	154 (10.9)	406 (14.1)	38 (10.7)	71 (10.2)	142 (12.6)	289 (13.1)
>3 – no (%)	85 (6.0)	148 (5.1)	15 (4.2)	34 (4.9)	68 (6.0)	114 (5.2)
Smoking						
1st Antenatal visit – no. (%)	139 (10.4)	258 (9.5)	38 (11.2)	71 (10.7)	156 (14.6)	283 (13.5)
Missing	75 (5.3)	147 (5.1)	15 (4.2)	33 (4.7)	61 (5.4)	105 (4.8)
Before pregnancy – no. (%)	225 (20.1)	329 (16.9)	89 (26.4)	111 (22.2)	242 (26.5)	306 (19.9)
Missing	293 (20.7)	926 (32.2)	17 (4.8)	194 (27.9)	215 (19.0)	666 (30.2)
Mode of delivery						
Vaginal – no. (%)	1112 (79.7)	2273 (81.7)	265 (75.3)	563 (83.3)	853 (77.3)	1704 (79.8)
Assisted vaginal – no. (%)	79 (5.7)	197 (7.1)	34 (9.7)	33 (4.9)	67 (6.1)	115 (5.4)
Emergency cesarean – no. (%)	89 (6.4)	146 (5.2)	25 (7.1)	38 (5.6)	67 (6.1)	153 (7.2)
Planned cesarean – no. (%)	116 (8.3)	168 (6.0)	28 (8.0)	42 (6.2)	116 (10.5)	163 (7.6)

ED, Eating Disorder; yr, year; BMI, Body Mass Index; SD, Standard Deviation; IQR, Interquartile Range

© 2022 Mantel Ä et al. *JAMA Network Open*.

eTable 4. Parental Sociodemographic Factors and Psychiatric Comorbidities of Exposed Children of Mothers With Eating Disorder and Their Full Maternal Cousins

Characteristics	Children exposed to maternal Anorexia Nervosa N 1412	Unexposed Comparator children N 3878	Children exposed to maternal Bulimia Nervosa N 354	Unexposed Comparator children N 695	Children exposed to maternal Unspecified ED N 1128	Unexposed Comparator children N 2201
**MATERNAL**						
Educational level						
< 9 yr – no. (%)	100 (7.1)	223 (7.8)	31 (8.8)	44 (6.3)	138 (12.2)	187 (8.5)
9-12 yr – no. (%)	528 (37.4)	1107 (38.5)	141 (39.8)	280 (40.3)	497 (44.1)	945 (42.9)
>12 yr – no. (%)	757 (53.6)	1529 (53.1)	179 (50.6)	353 (50.8)	475 (42.1)	1040 (7.3)
Missing	27 (1.9)	19 (0.7)	3 (0.9)	18 (2.6)	18 (1.6)	29 (1.3)
Psychiatric comorbidities †						
Depressive disorder – no. (%)	271 (19.2)	109 (3.8)	164 (46.3)	31 (4.5)	405 (35.9)	62 (2.8)
Anxiety disorder – no. (%)	185 (13.1)	98 (3.4)	87 (24.6)	19 (2.7)	272 (24.1)	82 (3.7)
ADHD – no. (%)	19 (1.4)	5 (0.2)	7 (2.0)	1 (0.1)	22 (2.0)	11 (0.5)
ASD – no. (%)	9 (0.6)	1 (0.03)	3 (0.9)	1 (0.1)	9 (0.8)	0
Substance abuse – no. (%)	75 (5.3)	30 (1.0)	33 (9.3)	8 (1.2)	101 (9.0)	22 (1.0)
**PATERNAL**	**1399 (99.1)***	**2848 (99.0)***	**352 (99.4)***	**691 (99.4)***	**1106 (98.1)***	**2176 (98.9)***
Age, mean yr ± SD	32.7 ± 6.1	31.7 ± 5.3	32.6 ± 5.8	31.3 ± 5.5	32.5 ±6.1	31.6 ± 5.9
Educational level						
< 9 yr – no. (%)	131 (9.4)	252 (9.1)	27 (7.7)	64 (9.3)	113 (10.2)	249 (11.4)
9-12 yr – no. (%)	658 (47.0)	1438 (50.5)	185 (52.6)	364 (52.7)	555 (50.2)	1045 (48.0)
>12 yr – no. (%)	583 (41.7)	1066 (37.4)	135 (38.4)	237 (34.3)	399 (36.1)	793 (36.4)
Missing	27 (1.9)	85 (3.0)	5 (1.4)	26 (3.8)	39 (3.5)	89 (4.1)
Psychiatric comorbidities †						
Depressive disorder – no. (%)	35 (2.5)	20 (0.7)	11 (3.1)	6 (0.9)	47 (4.3)	21 (1.0)
Anxiety disorder – no. (%)	30 (2.1)	33 (1.2)	9 (2.6)	7 (1.0)	20 (1.8)	8 (0.4)
ADHD – no. (%)	10 (0.7)	2 (0.1)	0	1 (0.1)	12 (1.1)	4 (0.2)
ASD – no. (%)	0	0	1 (0.3)	0	2 (0.2)	0
Substance abuse – no. (%)	24 (1.7)	23 (0.8)	9 (2.6)	1 (0.1)	23 (2.1)	26 (1.2)

ED, Eating disorder; ADHD, Attention-deficit hyperactivity disorder; ASD Autism spectrum disorder.
*Number (%) of study subjects with valid information on father personal identification number. †Registered diagnosis in the patient register prior to delivery date.

© 2022 Mantel Ä et al. *JAMA Network Open*.

eTable 5. Relative Risk of Neurodevelopmental Disorders Among Children of Mothers With Unspecified Eating Disorder Without Overlapping Diagnoses

	ATTENTION DEFICIT HYPERACTIVITY DISORDER					AUTISM SPECTRUM DISEASE				
	Rate /1000 person-yr (95% CI)		Hazard ratio (95% CI)			Rate /1000 person-yr (95% CI)		Hazard ratio (95% CI)		
**Unspecified ED**	**Maternal ED**	**Maternal Non-ED**	**HR_{Crude}***	**$HR_{Adjusted1}$‡**	**$HR_{Adjusted2}$α**	**Maternal ED**	**Maternal Non-ED**	**HR_{Crude}***	**$HR_{Adjusted1}$‡**	**$HR_{Adjusted2}$α**
**All**	9.1 (5.4-12.8)	4.5 (3.6-5.5)	1.89 (1.59-2.26)	1.49 (1.19-1.87)	1.52 (1.21-1.91)	1.9 (0.2-3.6)	1.2 (0.7-1.7)	1.52 (1.09-2.13)	1.12 (0.71-1.76)	1.10 (0.69-1.75)
**Ongoing**	11.4 (2.5-20.3)	4.5 (3.6-5.5)	2.29 (1.70-3.07)	1.58 (1.05-2.37)	1.67 (1.12-2.48)	2.6 (-1.6-6.9)	1.2 (0.7-1.7)	2.09 (1.18-3.70)	0.95 (0.41-2.18)	1.04 (0.45-2.43)
**Previous**	8.5 (4.4-12.6)	4.5 (3.6-5.5)	1.78 (1.44-2.18)	1.54 (1.20-1.98)	1.55 (1.20-2.01)	1.8 (-0.1-3.6)	1.2 (0.7-1.7)	1.37 (0.92-2.03)	1.03 (0.63-1.70)	1.02 (0.61-1.71)

*Crude hazard ratio adjusted for maternal age at birth, sex, birth year, maternal smoking status and parity

‡Adjusted hazard ratio adjusted for maternal educational level and psychiatric comorbidities (anxiety disorder, depressive disorder, ADHD, ASD) and alcohol or substance use disorder in addition to factors adjusted for in crude model

αAdjusted hazard ratio adjusted for paternal educational level and psychiatric comorbidities (anxiety disorder, depressive disorder, ADHD, ASD) and alcohol or substance use disorder in addition to factors adjusted for in $HR_{adjusted1}$-model

© 2022 Mantel Ä et al. *JAMA Network Open*.

eTable 6. Relative Risk of Neurodevelopmental Disorders Among Children of Mothers With Eating Disorder Stratified by Sex

	**ATTENTION DEFICIT HYPERACTIVITY DISORDER**					**AUTISM SPECTRUM DISEASE**				
	**Rate /1000 person-yr (95% CI)**		**Hazard ratio (95% CI)**			**Rate /1000 person-yr (95% CI)**		**Hazard ratio (95% CI)**		
	**Maternal ED**	**Maternal Non-ED**	**HR_{Crude}***	**$HR_{Adjusted1}$ ‡**	**$HR_{Adjusted2}$ α**	**Maternal ED**	**Maternal Non-ED**	**HR_{Crude}***	**$HR_{Adjusted1}$ ‡**	**$HR_{Adjusted2}$ α**
**Anorexia nervosa**										
**Female**	4.5 (1.6-7.5)	2.8 (1.8-3.9)	1.61 (1.26-2.06)	1.44 (1.09-1.90)	1.46 (1.10-1.95)	1.4 (-0.3-3.0)	0.6 (0.1-1.1)	2.42 (1.54-3.80)	2.28 (1.34-3.85)	2.24 (1.31-3.82)
**Male**	8.7 (4.6-12.7)	6.6 (5.0-8.2)	1.31 (1.09-1.56)	1.22 (1.00-1.49)	1.17 (0.95-1.44)	2.9 (0.6-5.3)	1.6 (0.8-2.3)	1.90 (1.41-2.54)	1.81 (1.29-2.53)	1.87 (1.33-2.64)
**Bulimia nervosa**										
**Female**	5.1 (-0.8-10.8)	2.9 (1.0-4.9)	1.72 (1.02-2.89)	1.60 (0.81-3.17)	1.56 (0.78-3.10)	1.3 (-1.0-4.3)	0.7 (-0.3-1.7)	1.73 (0.69-4.35)	2.08 (0.55-7.89)	2.24 (0.60-8.46)
**Male**	13.7 (4.3-23.1)	7.0 (4.0-10.0)	1.98 (1.43-2.75)	1.41 (0.91-2.19)	1.48 (0.96-2.30)	4.7 (-0.8-10.2)	1.5 (0.1-2.9)	3.16 (1.84-5.41)	1.41 (0.59-3.36)	1.36 (0.57-3.24)
**Unspecified ED**										
**Female**	6.1 (2.4-9.7)	3.2 (2.0-4.4)	1.92 (1.50-2.46)	1.40 (1.00-1.96)	1.38 (0.98-1.95)	1.3 (-0.4-2.9)	0.8 (0.2-1.3)	1.68 (1.04-2.73)	1.25 (0.62-2.52)	1.20 (0.59-2.46)
**Male**	11.7 (6.8-16.9)	5.9 (4.3-7.5)	2.04 (1.71-2.44)	1.77 (1.40-2.25)	1.75 (1.37-2.24)	3.3 (0.7-6.0)	1.6 (0.8-2.4)	2.07 (1.52-2.81)	1.48 (0.95-2.31)	1.51 (0.96-2.36)

*Crude hazard ratio adjusted for maternal age at birth, sex, birth year, maternal smoking status and parity

‡Adjusted hazard ratio adjusted for maternal educational level and psychiatric comorbidities (anxiety disorder, depressive disorder, ADHD, ASD) and alcohol or substance use disorder in addition to factors adjusted for in crude model

αAdjusted hazard ratio adjusted for paternal educational level and psychiatric comorbidities (anxiety disorder, depressive disorder, ADHD, ASD) and alcohol or substance use disorder in addition to factors adjusted for in $HR_{adjusted1}$-model

© 2022 Mantel Ä et al. *JAMA Network Open*.

eTable 7. Relative Risk of Neurodevelopmental Disorders Among Children of Mothers With Eating Disorder Stratified by Birth Cohort

	ATTENTION DEFICIT HYPERACTIVITY DISORDER					AUTISM SPECTRUM DISEASE				
	Rate /1000 person-yr (95% CI)		Hazard ratio (95% CI)			Rate /1000 person-yr (95% CI)		Hazard ratio (95% CI)		
	Maternal ED	Maternal Non-ED	HR_{Crude}*	$HR_{Adjusted1}$ ‡	$HR_{Adjusted2}$ α	Maternal ED	Maternal Non-ED	HR_{Crude}*	$HR_{Adjusted1}$ ‡	$HR_{Adjusted2}$ α
**Anorexia nervosa**										
**Birth year <2007**	7.3 (3.5-11.1)	5.1 (3.7-6.6)	1.41 (1.19-1.68)	1.36 (1.14-1.66)	1.36 (1.11-1.65)	2.2 (0.1-4.3)	1.2 (0.5-1.8)	1.91 (1.41-2.57)	2.00 (1.43-2.76)	2.00 (1.43-2.78)
**Birth year ≥2007**	5.1 (2.0-8.2)	3.8 (2.6-5.0)	1.33 (0.99-1.80)	1.04 (0.73-1.48)	0.99 (0.69-1.42)	2.1 (0.1-4.1)	0.9 (0.3-1.4)	2.40 (1.53-3.78)	1.70 (0.92-3.16)	1.78 (0.93-3.41)
**Bulimia nervosa**										
**Birth year <2009**	11.7 (2.1-21.3)	6.8 (3.5-10.1)	1.74 (1.25-2.41)	1.36 (0.92-2.01)	1.35 (0.91-2.01)	3.8 (-1.7-9.3)	1.3 (-0.1-2.7)	2.93 (1.70-5.06)	1.36 (0.91-2.01)	1.35 (0.91-2.01)
**Birth year ≥2009**	6.1 (0.2-11.9)	2.4 (0.8-4.1)	2.58 (1.50-4.40)	1.98 (0.84-4.66)	2.16 (0.91-5.11)	2.0 (-1.3-5.3)	0.9 (-0.1-1.9)	2.57 (1.50-4.40)	1.98 (0.84-4.66)	2.16 (0.91-5.11)
**Unspecified ED**										
**Birth year <2007**	9.3 (4.2-6.8)	5.1 (3.4-6.8)	1.83 (1.53-2.20)	1.65 (1.32-2.06)	1.59 (1.26-2.01)	2.1 (-0.3-4.6)	1.2 (0.4-2.0)	1.81 (1.27-2.58)	1.65 (1.32-2.06)	1.59 (1.26-2.01)
**Birth year ≥2007**	8.5 (4.6-12.3)	3.6 (2.5-4.7)	2.38 (1.86-3.04)	1.69 (1.16-2.46)	1.71 (1.17-2.50)	2.6 (0.4-4.7)	1.2 (0.6-1.8)	2.38 (1.86-3.04)	1.69 (1.16-2.46)	1.71 (1.17-2.50)

*Crude hazard ratio adjusted for maternal age at birth, sex, birth year, maternal smoking status and parity

‡Adjusted hazard ratio adjusted for maternal educational level and psychiatric comorbidities (anxiety disorder, depressive disorder, ADHD, ASD) and alcohol or substance use disorder in addition to factors adjusted for in crude model

αAdjusted hazard ratio adjusted for paternal educational level and psychiatric comorbidities (anxiety disorder, depressive disorder, ADHD, ASD) and alcohol or substance use disorder in addition to factors adjusted for in $HR_{adjusted1}$-model

© 2022 Mantel Ä et al. *JAMA Network Open*.

eReferences

1. Ludvigsson JF, Otterblad-Olausson P, Pettersson BU, Ekbom A. The Swedish personal identity number: possibilities and pitfalls in healthcare and medical research. European journal of epidemiology. 2009;24(11):659-67.
2. Ludvigsson JF, Andersson E, Ekbom A, Feychting M, Kim JL, Reuterwall C, et al. External review and validation of the Swedish national inpatient register. BMC public health. 2011;11:450.

© 2022 Mantel Ä et al. *JAMA Network Open*.
